# Mitophagy promotes replication of oncolytic Newcastle disease virus by blocking intrinsic apoptosis in lung cancer cells

**DOI:** 10.18632/oncotarget.2219

**Published:** 2014-07-15

**Authors:** Gang Meng, Mao Xia, Diancheng Wang, Aiping Chen, Yongshan Wang, Hongwei Wang, Decai Yu, Jiwu Wei

**Affiliations:** ^1^ Jiangsu Key Laboratory of Molecular Medicine, Medical School of Nanjing University, Nanjing, China; ^2^ The Affiliated Drum Tower Hospital, Medical School of Nanjing University, Nanjing, China; ^3^ Nanjing University Hightech Institute at Suzhou, Suzhou, China; ^4^ Institute of Veterinary Medicine, Jiangsu Academy of Agricultural Sciences, Nanjing, China

**Keywords:** Newcastle disease virus, mitophagy, apoptosis, autophagy, cancer

## Abstract

Apoptosis contributes to antitumor effect of Newcastle disease virus (NDV). Autophagy is a protective response under cellular stress including viral infection. How autophagy interferes with oncolysis of NDV remains unclear. In this study, we found that NDV La Sota strain induced autophagy and preserved autophagic flux in non-small cell lung cancer cells. NDV-induced autophagy promoted viral replication by blocking cancer cells from caspase-dependent apoptosis. Moreover, we found that NDV recruited SQSTM1-mediated mitophagy to control cytochrome c release, and thus blocked intrinsic pro-apoptotic signaling. Finally, we observed an enhanced oncolysis in NSCLC cells treated with NDV in the presence of an autophagy inhibitor 3-methyladenine (3-MA). Interestingly, a more profound antitumor effect could be achieved when administration of 3-MA was postponed to 24 h after NDV infection. Our findings unveil a novel way that NDV subverts mitophagy to favor its replication by blocking apoptosis, and provide rationale for systemic therapeutic cohort combining NDV with autophagy inhibitors in cancer therapy.

## INTRODUCTION

Understanding oncolytic mechanisms in cancer cells is crucial for further improvement of oncolytic virotherapy. Newcastle disease virus (NDV) is a single-stranded, negative-sense, non-segmented RNA virus, a member of the Avulavirus genus in the family *Paramyxoviridae*. NDV possesses oncolytic properties and has been investigated in clinical studies with good safety records [1, 2]. Studies show that apoptosis dominantly contributes to NDV-induced cell death [3, 4]. Both extrinsic and intrinsic apoptotic pathways can be activated in cancer cells after infection with NDV including Beaudette C and La Sota strains [4]. It seems that the intrinsic apoptotic pathway is more important in NDV-mediated cell death, as infection of NDV in a variety of tumor cell lines leads to loss of mitochondrial membrane potential and activation of caspase9 [4]. It has been shown that the virus-specific HN protein causes apoptosis by a yet uncharacterized mechanism in chicken embryo fibroblast cells [1, 5], and that NDV-induced apoptosis in cancer cells is independent of type I IFN signaling [4, 6]. Interestingly, several previous studies have demonstrated that the preferential antitumor activity of NDV is not due to impaired antiviral innate immune responses in tumor cells, in which robust type I IFN responses have been observed after viral infection [7-9]. Some recent reports show that the oncolytic NDV preferentially replicates in apoptosis-resistant cells overexpressing Bcl-X_L_ or Livin, and exerts a more profound antitumor effect [6, 10].

Accumulated evidences show that autophagy may counteract with apoptosis [11-14]. Autophagy is a conserved homeostatic process for eukaryotic cell under metabolic stress or pathogens infection. The autophagic flux is described as a process, by which a portion of cytoplasm is enclosed by isolated membrane to form autophagosome followed by fusion with lysosome to form autolysosome, where the sequestered contents are degraded [15, 16]. Autophagy eliminates damaged or redundant intracellular components, such as unfolded proteins, dysfunctional organelles or viral pathogens, etc. [17-19]. Mitophagy is a type of selective macroautophagy and is a specific autophagic elimination of damaged mitochondria [19]. Recent works reveal that SQSTM1 (sequestosome-1, also known as p62) acts as a signaling hub through its ability to regulate the packing and delivery of polyubiquitinated, misfolded proteins and dysfunctional organelles for their clearance through autophagy in mammalian cells [20-23].

Some experimental evidences show that autophagy is an essential host defense response to fight infection by destroying infectious pathogens trapped within autophagosomes, and plays an important role in the induction of both innate and adaptive immune response [14, 18, 24-26], whereas some studies show that viruses also subvert autophagy to enhance viral replication and release, such as hepatitis C virus, dengue virus, and measles virus [27-31]. It has been shown recently that NDV strain Beaudette C triggers autophagy both in human U251 glioma cells and in chicken cells leading to enhanced virus replication [32, 33]. However, it remains unclear how autophagy promotes viral replication. In this study, we investigated how NDV La Sota strain recruits autophagy to counteract with apoptosis and provided the potential antitumor strategy by combining NDV with autophagy interference.

## RESULTS

### NDV infection induces autophagosomal accumulation and preserves autophagic flux

**Figure 1:**
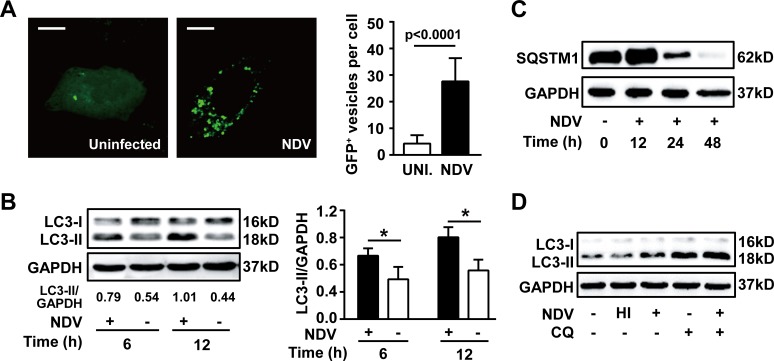
NDV infection induces autophagy and preserves autophagic flux (A) A549 lung cancer cells were transiently transfected with EGFP-LC3 plasmid for 24 h followed by infection with NDV-La Sota (10 HAU/10^6^ cells), or left uninfected for another 12 h. Aggregation of EGFP-LC3 at autophagosomes (EGFP^+^ vesicles, white dots) was monitored by confocal microscopy (left panel) and the dots per cell were counted (right panel). Bars represent 10 μm. Results are means + SD of 30 cells. (B) Levels of lipidated LC3 (LC3-II) were detected by western blot in cell lysates obtained from A549 cells infected with NDV (10 HAU/10^6^ cells) or left uninfected for 6 and 12 h (left panel). LC3-II/GAPDH ratio was quantified by densitometric analysis (right panel). Means + SD of three independent experiments are shown. * p< 0.05. (C) Degradation of SQSTM1 was monitored by western blot in cell lysates obtained from A549 cells infected with NDV (10 HAU/10^6^ cells) for 0, 12, 24 and 48 h. Blots are representative of two independent experiments. (D) A549 cells were infected with NDV (10 HAU/10^6^ cells) for 12 h followed by chloroquine treatment for another 6 h. Lipidated LC3 was determined by western blot. Uninfected cells or cells infected with heat inactivated NDV (HI) were used as controls. A representative blot from two independent experiments is shown.

It has been shown that NDV Beaudette C strain induces autophagy both in U251 glioma and chicken cells. To know whether NDV La Sota strain also elicits such responses, autophagic processes was determined in human non-small cell lung cancer A549 cells. We found that cellular LC3 accumulated to form autophagosomes (Fig. 1A) and lipidation of LC3 was robustly induced following NDV infection (Fig. 1B). In addition, we found that SQSTM1, a crucial adaptor protein cargos contents to autophagosomes for autolysosomal degradation, was markedly reduced 24 h and almost erased 48 h after NDV infection (Fig. 1C), suggesting that the autophagic flux is preserved following NDV infection. This was further confirmed by the fact that lapidated LC3 was massively increased in the presence of chloroquine, an inhibitor of lysosomal degradation (Fig. 1D). Interestingly, lipidation of LC3 was not induced by heat-inactivated NDV (Fig. 1D), suggesting that active viral replication is required for autophagy induction. Taken together, these results demonstrate that NDV infection induces a completed autophagic flux in A549 cells.

### Autophagy mitigates NDV-induced apoptotic cell death in NSCLC

**Figure 2:**
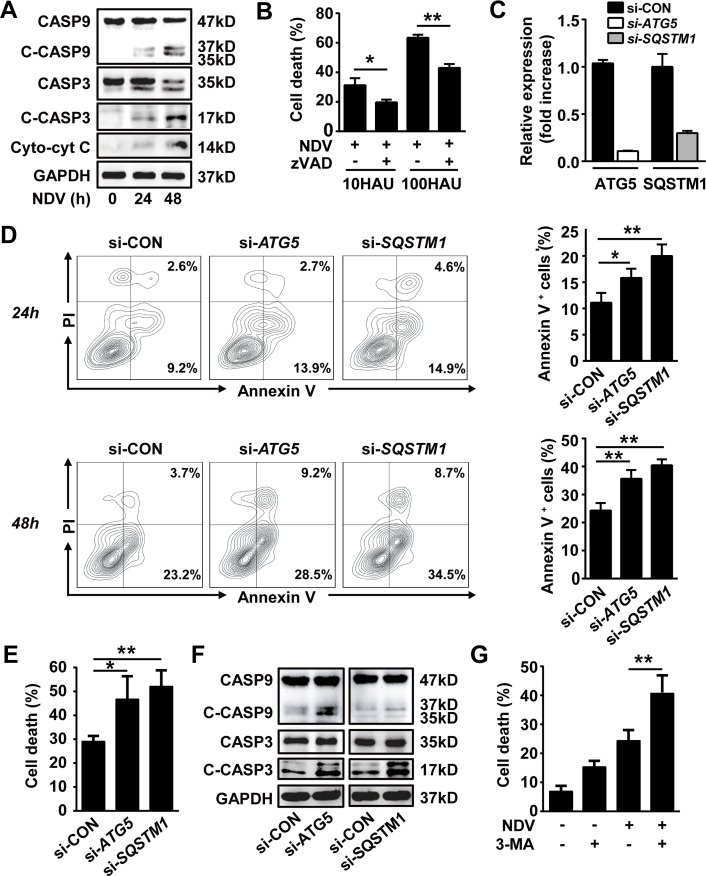
Autophagy blocks NDV-induced apoptosis (A) A549 cells were infected with NDV (10 HAU/10^6^ cells) for 0, 24 or 48 h, cell lysates were then harvested to determine the cleaved caspase-3 and -9 by western blot. Or cell lysates were fractionated to determine cytoplasm cytochrome c by western blot. Blots are representative of two independent experiments. (B) A549 cells were pretreated with 80 μM z-VAD-fmk for 2 h followed by NDV infection with a dose of 10 or 100 HAU per 10^6^ cells for another 48 h. Cell death was measured by trypan blue exclusion. Means + SD of triplicates are shown, similar results were obtained in two independent experiments. (C & D) A549 cells were transfected with siRNAs against *ATG5* or *SQSTM1*, or with non-specific control siRNA for 24 h followed by infection with NDV (10 HAU/10^6^ cells) for another 24 or 48 h. (C) gene silencing efficacy was evaluated by qRT-PCR, and (D) cells were stained by annexin V and PI before subjected to flow cytometry. Annexin V positive cells were counted as apoptotic cells (left panel). Means + SD of triplicates are shown (right panel). Similar results were obtained in two independent experiments. (E) A549 cells were transfected with siRNAs targeting *ATG5, SQSTM1*, or non-specific siRNA followed by infection with NDV (10 HAU/10^6^ cells) for 48 h. Cell death was determined by trypan blue exclusion. Means + SD of triplicates are shown. Similar results were obtained in two independent experiments. (F) A549 cells were transfected with siRNAs targeting *ATG5, SQSTM1*, or non-specific siRNA for 24 h followed by NDV infection (10 HAU/10^6^ cells) for another 24 h, Cleaved caspase-3 and -9 were then detected by western blot. Blots are representative of two independent experiments. (G) A549 cells were infected with NDV in the presence or absence of 5 mM 3-methyladenine (3-MA), and cell death was determined by trypan blue exclusion. Similar results were obtained in 3 independent experiments. * p < 0.05, ** p < 0.01.

We next investigated the role of autophagy in NDV-induced apoptosis. First we confirmed that NDV infection activates intrinsic pro-apoptotic signaling in A549 lung cancer cells, as cytoplasmic cytochrome c release, cleaved capase-9 and -3 were increased after NDV infection in a time-course manner (Fig. 2A). This was further confirmed by that the cell death was significantly decreased in the presence of a pan-caspase inhibitor z-VAD-fmk (Fig. 2B). We then evaluated the apoptosis in lung cancer cells with impaired autophagy. By silencing with siRNAs targeting autophagy related genes ATG5, a key protein for initiation of pre-autophagosomal membrane formation in mammalians, and SQSTM1 (Fig. 2C), we found that the annexin-V positive apoptotic cells were significantly increased both at early and late stage following NDV infection in autophagy-impaired cells (Fig. 2D). This suggests that autophagy counteracts apoptosis after NDV infection. The protective effect of autophagy in NDV infected cells was further evidenced by trypan blue exclusion (Fig. 2E). In line, the cleaved pro-apoptotic proteins caspase-9 and -3 were also enhanced in autophagy-impaired cells after NDV infection (Fig. 2F). Moreover, we further confirmed that NDV-induced cell death was significantly increased in the presence of an autophagy inhibitor 3-methyladenine (3-MA). Taken together, these results show that NDV induces apoptotic cell death and that autophagy plays a protective role against apoptosis.

### Autophagy promotes NDV replication by mitigating cell apoptosis

**Figure 3:**
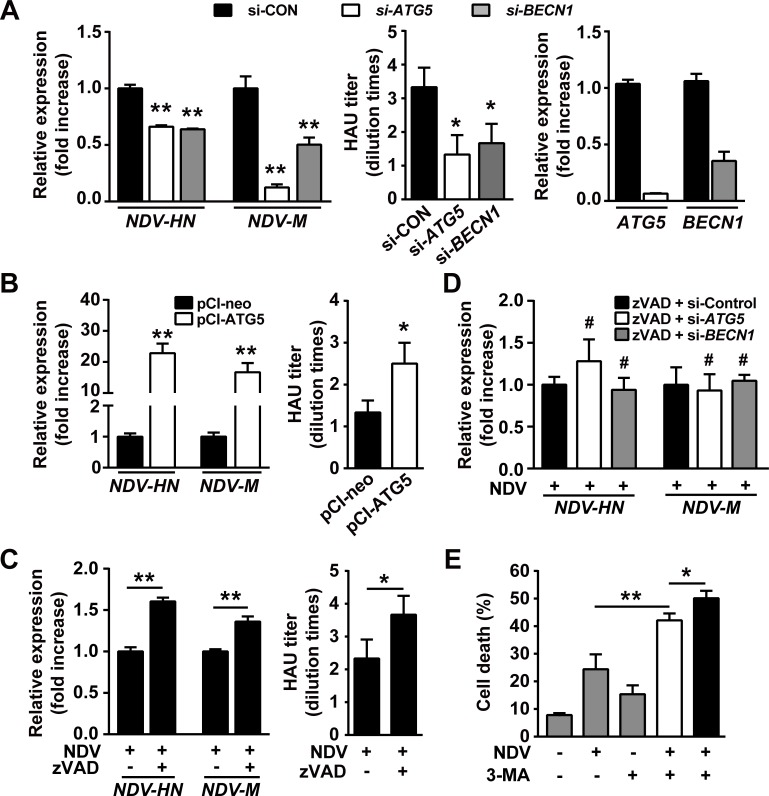
Autophagy promotes viral replication by mitigating apoptosis (A) A549 cells were transfected with siRNAs targeting *ATG5, BECN1*, or non-specific control siRNA for 24 h followed by NDV infection (10 HAU/10^6^ cells) for another 24 h. Replication of NDV was determined by qRT-PCR to quantify gene expression of *NDV-HN* and *-M* (left panel). Means + SD of quadruplicates are shown. Similar results were obtained in two independent experiments. Or viral particles were harvested by two rounds of freezing-thawing cycles and viral titer was measured by Hemagglutination Assay (middle panel). Means + SD of three independent experiments are shown. Quality control of gene silencing efficacy by siRNAs was monitored by qRT-PCR (right panel). (B) A549 cells were transfected with a plasmid expressing *ATG5* for 24 h followed by infection with NDV (10 HAU/10^6^ cells) for another 24 h, backbone vector pCI-neo was used as control. Replication of NDV was determined either by qRT-PCR to quantify *NDV-HN* and *-M* gene expression (left panel) or by Hemagglutination Assay to quantify relative viral particles (right panel). Means + SD are shown. Similar results were obtained in 2 independent experiments. (C) A549 cells were pretreated with 80 μM z-VAD-fmk for 2 h followed by NDV infection for 24 h at a dose of 10 HAU/10^6^ cells. Virus replication was analyzed either by qRT-PCR (left panel) or by Hemagglutination Assay (right panel). Means + SD of quadruplicates (left panel) or triplicates (right panel) are shown. Similar results were obtained in two independent experiments. (D) A549 cells were pretreated with 80 μM z-VAD-fmk for 2 h followed by transfection with siRNAs targeting *ATG5, BECN1*, or non-specific control siRNA for another 24 h. Cells were then infected with NDV (10 HAU/10^6^ cells) for another 24 h. Viral HN and M genes were quantified by qRT-PCR. Means + SD of quadruplicates are shown. Similar results were obtained in two independent experiments. (E) A549 cells were treated with 5 mM 3-MA either immediately (open bar) or 24 h (filled bar) after NDV infection (10 HAU/10^6^ cells). Cell death was determined by trypan blue exclusion 48 h after NDV infection. Cells treated with NDV or 3-MA alone (grey bars) were used as controls. Means + SD of triplicates are shown. Similar results were obtained in two independent experiments. * p < 0.05, ** p < 0.01.

Having shown that autophagy blocks cell apoptosis after NDV infection, we sought to investigate whether the attenuated apoptosis contributes to viral replication. We found that both viral structural gene expression and viral titer were significantly decreased in autophagy knockdown cells (Fig. 3A). For instance, the viral structural M gene was reduced to 50-80% in autophagy-impaired cells. Conversely, viral replication was markedly increased in cells overexpressing ATG5 gene, e.g. the expression of viral structural genes was robustly increased up to 20 folds compared to mock-transfected cells (Fig. 3B). These results indicate that autophagy favors NDV replication. Next we confirmed that viral replication could be enhanced by blocking apoptosis using a pan-caspase inhibitor z-VAD-fmk (Fig. 3C). Interestingly, we found that viral structural gene expression could not be decreased in autophagy knockdown cells in the presence of z-VAD-fmk (Fig. 3D). These results suggest that autophagy favors viral replication by preventing cell apoptosis.

As sufficient viral replication and efficient apoptotic induction are both required in NDV-based oncolysis, it seems that autophagy plays contradict roles in NDV-mediated oncolysis. On the one hand, autophagy promotes viral replication, on the other hand, autophagy compromises NDV-induced apoptosis. We next sought to find a solution for this paradox. As shown in Fig. 3E, while the oncolysis was enhanced in A549 cells treated simultaneously with NDV and 3-MA, a more profound oncolysis was achieved when 3-MA was administered 24 h after NDV infection (Fig. 3E). This result suggests that sophistic manipulation of autophagy is required for efficient NDV-based oncolysis.

### NDV infection subverts mitophagy to control cytochrome c release

**Figure 4:**
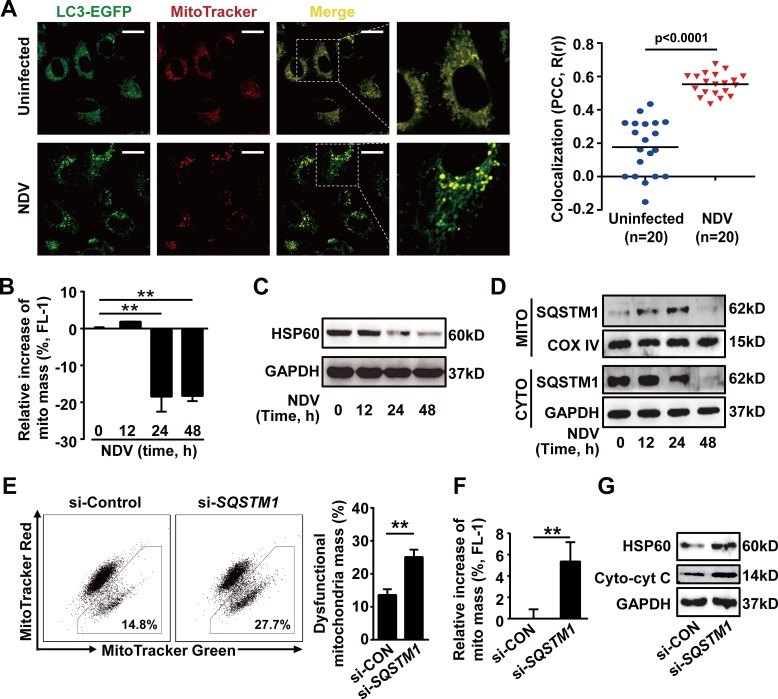
SQSTM1-mediated mitophagy controls cytochrome c release in NDV-infected cells (A) A549 cells were transiently transfected with a plasmid encoding EGFP-LC3 for 24 h followed by infection with NDV (10 HAU/10^6^ cells) for 12 h. Cells were then stained by MitoTracker Red before subjected to confocal microscopy. Co-localization (yellow dots) of mitochondria (red) with autophagosomes (green dots) are shown (left panel) and was quantified in twenty cells by calculating Pearson's correlation coefficient (PCC, R(r)) (right panel). (B) A549 cells were infected with NDV (10 HAU/10^6^ cells) for 12, 24, or 48 h. Cells were then stained by MitoTracker Green and the mitochondrial mass was analyzed by flow cytometry. Results are percentages of increased mitochondrial mass. Means + SD of three independent experiments are shown. (C) HSP60 expression was detected by western blot in A549 cells infected with NDV (10 HAU/10^6^ cells) for 12, 24 and 48 h. Blots are representative of two independent experiments. (D) Translocation changes of SQSTM1 was determined by western blot in mitochondria or cytoplasm fractionated from A549 cells infected with NDV for 0, 12, 24 and 48 h. COX IV and GAPDH were used as loading controls for mitochondrion and cytoplasm, respectively. Blots are representative of two independent experiments. (E) A549 cells were transfected with siRNA targeting with *SQSTM1*, or non-specific control siRNA for 24 h followed by NDV infection (10 HAU/10^6^ cells) for another 24 h. Cells were then stained by MitoTracker Red and MitoTracker Green, and the dysfunctional mitochondrial mass was quantified by flow cytometry. Dot plots of subpopulations are depicted (left panel) and percent of dysfunctional mitochondria was quantified ((total mitochondria (green) -functional mitochondria (red))/total mitochondria (green) × 100%, right panel). Means + SD of three independent experiments are shown. (F) A549 cells were transfected with SQSTM1 siRNA or with non-specific control siRNA for 24 h followed by NDV infection (10 HAU/10^6^ cells) for another 24 h, cells were then stained by MitoTracker Green and the total mitochondria mass was measured by flow cytometry. Relative increase of mitochondria was evaluated. Mean + SD of three independent experiments. ** p < 0.01. (G) A549 cells were transfected with SQSTM1 siRNA or non-specific control siRNA for 24 h followed by NDV infection (10 HAU/10^6^ cells) for another 24 h. HSP60 and cytoplasmic cytochrome c were determined by western blot. Blots are representative of two independent experiments.

Finally, we wanted to know the precise mechanisms by which NDV-induced autophagy counteracts apoptosis. We found that NDV infection induced mitophagy as evidenced by the increased co-localization of autophagosomes and mitochondria (Fig. 4A). This was further confirmed by the facts that mitochondrial mass was reduced (Fig. 4B) and that the conservative mitochondrial protein HSP60 was decreased (Fig. 4C) after NDV infection, as mitophagy is known to eliminate dysfunctional mitochondria. Moreover, we found that the SQSTM1 protein translocated from cytoplasm onto mitochondria, as evidenced by accumulation of SQSTM1 on mitochondria and concomitant reduction in cytoplasm 12 h and 24 h after NDV infection (Fig. 4D). The total SQSTM1 was exhausted 48 h post-infection (Fig. 4D). These results suggest that SQSTM1 is involved in mitophagy formation and mitophagic degradation after NDV infection. This was then confirmed by the increase of dysfunctional mitochondria (Fig. 4E), and total mitochondrial mass (Fig. 4F) in SQSTM1 knockdown cells. Finally, we found that impaired elimination of dysfunctional mitochondria in SQSTM1 knockdown cells led to increased cytochrome c release (Fig. 4G). Taken together, our data show that NDV infection utilizes SQSTM1-mediated mitophagy to control cytochrome c release, and thus blocks apoptosis.

## DISCUSSION

Our study characterizes for the first time that oncolytic NDV La Sota strain usurps SQSTM1-mediated mitophagy to promote viral replication by mitigating intrinsic pro-apoptotic cascades initiated by cytochrome c release. We also provide a novel strategy by manipulating autophagy to improve NDV-mediated oncolysis in lung cancer cells. Our work is important for future improvement of NDV-based oncolytic virotherapy.

We showed that NDV La Sota strain induced a complete autophagic flux, which promoted viral replication in lung cancer cells. A similar result was observed in the U251 glioma and chicken cells infected with NDV Beaudette C strain [32, 33]. However, the precise mechanisms by which autophagy promote viral replication remains unclear. We found that autophagy promoted viral replication by blocking caspase-dependent apoptosis, which is consistent with previous studies showing that apoptosis-resistant cancer cells favor NDV replication [6].

Previous studies have shown that NDV infection results in loss of mitochondrial membrane potential [1, 4, 6], and malfunction of the mitochondrial respiratory chain then increases cytochrome c release leading to caspase-dependent cell death [34, 35]. In this study, we found that mitophagy contributed to elimination of damaged mitochondria following NDV infection, which led to controlled cytochrome c release, therefore, blocked intrinsic proapoptotic cascades. Moreover, we found that NDV-induced mitophagy was mediated by SQSTM1/p62, a protein often overexpressed in many tumor types [36-39]. Given that SQSTM1-mediated mitophagy is required for preventing NDV-induced apoptosis and thus favors viral replication, it might explain why NDV preferentially replicates in cancer cells.

As sufficient viral replication and effective induction of cell death are both required by NDV-based oncotherapy, it seems that autophagy plays contradict roles in NDV-mediated oncolysis. On the one hand, autophagy promotes NDV replication, which favors oncolysis. On the other hand, autophagy/mitophagy counteracts NDV-induced intrinsic apoptotic pathway and thus compromised NDV-mediated oncolysis. In our settings, it is likely that the enhanced viral replication is a consequence of attenuated apoptosis by autophagy. Therefore, a sophistic manipulation of autophagy may determine the outcome of NDV-based oncotherapy. Indeed, while autophagy inhibitor enhanced oncolysis in NSCLCs infected with NDV, a more profound oncolysis was achieved by delayed administration of autophagy inhibitor after NDV infection. This therapeutic cohort initially allows sufficient viral replication for a period of time, and subsequently induces effective apoptosis by blocking autophagy using an autophagy inhibitor.

In conclusion, our results clarify the mechanisms by which autophagy facilitates viral replication and provide rationale for systemic therapeutic cohort combining NDV with autophagy interference. This requires further preclinical investigations and might be useful for clinical trial design to improve NDV-based virotherapy.

## METHODS

### Cells, antibodies and reagents

Human non-small cell lung cancer cell line A549 (CCL-185) was obtained from Chinese Academy of Sciences Cell Bank of Type Culture Collection (CBTCCCAS) and cultivated in Dulbecco's Modified Eagle Medium (DMEM) supplemented with 5% fetal bovine serum, 2 mM L-glutamine, 100 U/L penicillin and 0.1 mg/ml streptomycin (all from Life technology, Grand Island, NY) and maintained in a humidified incubator with 5% CO_2_ at 37°C.

Antibodies used in this study were: anti-LC3 (Thermo Scientific, Waltham, MA, PAI-16930, 1:500 dilution), anti-p62/SQSTM1 (Epitomics, Burlingame, CA, #3340-1, 1:3000 dilution), anti-HSP60 (Epitomics, #1724-1, 1:10000 dilution), anti-caspase-3 (Cell Signaling Technology, #9662, 1:1000 dilution), anti-caspase-9 (Cell Signaling Technology, #9502, 1:1000 dilution), anti-cytochrome c (Epitomics, #2119-1, 1:1000 dilution), anti-HSP60 (Epitomics, #1724-1, 1:3000 dilution), anti-GAPDH (Bioworld, Minneapolis, MN, MB001, 1:5000 dilution) and HRP-conjugated secondary antibodies (Multisciences, Hangzhou, China, GAR007 and GAM007, 1:5000 dilution).

The following reagents, 3-Methyladenine (3-MA, #M9281), chloroquine (CQ, #C6628), z-VAD-fmk (#V116) and trypan blue (#T6146) were all obtained from Sigma-Aldrich (Saint Louis, MO).

### Plasmids, siRNAs and transfections

The siRNA targeting *ATG5* (Invitrogen, HS114104), *Beclin1/BECN1* (Invitrogen, HSS112731), *SQSTM1* (Invitrogen, HSS121770) and negative control siRNA (Invitrogen, 12935400) were all purchased from Invitrogen Stealth RNAi collection. pCI-neo-hATG5-HA (Addgene, Cambridge, MA, #22948) was provided by Noboru Mizushima (Tokyo Medical and Dental University, Tokyo, Japan). pCI-neo was obtained from Promega (Madison, WI, #1841). pBABEpuro-EGFP-LC3 (Addgene, #22405) was provided by Jayanta Debnath (University of California, San Francisco, CA, USA). 100 nM of siRNA or 500 ng/ml expression plasmids coupled with Lipofectamine 2000 (Invitrogen, 11668-019) were used for transfection of A549 on a 6- or 12-well plate according to the manufacturer's instructions. For all experiments, NDV infection was performed 24 h after siRNA transfection.

### NDV propagations and infections

NDV La Sota strain was obtained from Jiangsu Academy of Agricultural Sciences (JAAS, Jiangsu province of P.R.China), propagated in 9-day-old SPF embryonated chicken eggs from seed virus, harvested from the allantoic fluid and purified centrifugation at 3000 rpm for 10min. The viral particles in the supernatant were collected and cryopreserved at −80°C. The virus titer was determined by the hemagglutination test in which 1 hemagglutination unit (HAU) is defined as the lowest virus concentration leading to visible chicken erythrocyte agglutination. Briefly, 50 μl aseptic PBS was added to each well of a round-bottomed 96-well dish, mixed with 50 μl viral dilution or infected-cell lysate supernatant in the first column, then repeat mixing and transferring 50 μl to the next well, discard 50 μl from the last well into the bleach solution. Then 50 μl of 0.5% red blood cell working solution was added into each well, mix gently and left at room temperature for 45 minutes. The dilution times leading to visible erythrocyte agglutination was recorded.

Tumor cells were washed once by PBS and infected with NDV in empty DMEM at a dilution of 10 HAU/10^6^ cells for 3 h, and then completed medium was added in each well.

### Western blot analysis

Cells were lysed in RIPA buffer containing a protease inhibitor cocktail (Roche, Mannheim, Germany, 11873580001). Protein concentration was determined. Equal amounts of protein were separated by SDS-PAGE and electrophoretically transferred onto a PVDF membrane (Roche, 03010040001). After blocking with 5% nonfat milk in Tris-buffered saline containing 0.1% Tween-20 the membrane was incubated with specific primary antibodies, followed by incubation with appropriate horseradish peroxidase–conjugated secondary antibodies. Signals were detected using an enhanced chemiluminescence reagent (Millipore, Darmstadt, Germany, WBKLS0500) and subjected to Alpha Innotech Flour Chem-FC2 imaging system (Alpha Innotech, San Leanardo, CA).

### Quantitative RT-PCR

For quantitative reverse transcription-polymerase chain reaction (qRT-PCR), total cellular RNA was extracted with TRIZOL (Invitrogen, 15596-026) and RNA was reverse-transcribed (TaKaRa, Shiga, Japan, DRR036A). qPCR was performed using the Real-Time PCR system (ABI 7300, Advanced Biosystems, Foster, CA). Gene expression was calculated with the comparative Ct method and normalized to the endogenous levels of GAPDH.

Primer sequences used for qRT-PCR are as follows: *GAPDH*-forward 5'-CCATGTTCGTCATGGGTGTGAACCA-3', reverse 5'-GCCAGTAGAGGCAGGGATGATGTTC-3'; *NDV-HN*-forward 5'-GGGGGATAGGCAAAGAACTCATT-3', reverse 5'-GTATTGGCCGTCGAACCCTAAC-3'; *NDV-M*-forward 5'-AGTGATGTGCTCGGACCTTC-3', reverse 5'-CCTGAGGAGAGGCATTTGCTA-3'; *IFN-β*-forward 5'-CTTGGATTCCTACAAAGAAGC-3', reverse 5'-CATCTCATAGATGGTCAATGC-3'; *IP-10*-forward 5'-CTTCCAAGGATGGACCACACA-3', reverse 5'-CCTTCCTACAGGAGTAGTAGCAG-3'; *ATG5*-forward 5'-AAGCAACTCTGGATGGGATT-3', reverse 5'-GCAGCCACAGGACGAAAC-3'; *Beclin1*-forward 5'-GGATGGATGTGGAGAAAGGCAAG-3', reverse 5'-TGAGGACACCCAAGCAAGACC-3'; *SQSTM1*-forward 5'-GAACTCCAGTCCCTACAGAT-3', reverse 5'-CGATGTCATAGTTCTTGGTC-3'.

### Fluorescence microscopy

The pBABEpuro-EGFP-LC3 plasmid was transiently transfected in A549 cells 24 h prior to virus infection. Cells were stained with MitoTracker Red (Invitrogen, M7512) at a concentration of 100 nM for 20 minutes at 37°C and then fixed with 4% paraformaldehyde. Cells were observed under FLUOVIEW FV10i confocal microscope (Olympus, Tokyo, Japan) and images were analyzed using FV10-ASW 4.0 Viewer (Olympus).

### Cell viability assay

Cell death was determined by trypan blue exclusion assay. Cells were harvested by trypsin/EDTA (Life technology, Grand Island, NY) and then stained with 0.4% trypan blue staining solution for 5 min. Total cells were detected by Automated cell counter (Countstar, Inno-Alliance Biotech Inc., Wilmington, USA). Cell death (%) was counted as dead cells /total cell numbers × 100%.

### Flow cytometry

Apoptotic cell death was detected by Annexin V/propidium iodide (PI) staining assay (Invitrogen, V13241) according to the manufacturer's protocols. Briefly, cells were harvested and washed once with PBS, then resuspended in 100 μl binding buffer followed by incubation with 2.5 μl Annexin V per test for 20 min. Then 1 μl PI per test was added and then cells were analyzed by a FACSCalibur (Becton, Dickinson and Company, USA).

Dysfunctional mitochondria were monitored by fluorescence levels upon staining with 100 nM MitoTracker Green FM (total mitochondria) and 400 nM MitoTracker Red CMXRos (functional mitochondria, both from Invitrogen, M7514 and M7512) for 25 min at 37°C. Cells were then washed with PBS and analyzed at FL-1 and FL-3 by flow cytometry.

Mitochondrial mass was measured by fluorescence levels upon staining with 100 nM Mitotracker Green FM for 25 min at 37°C. Cells were then washed with PBS and analyzed at FL-1 by flow cytometry.

All data were analyzed using FlowJo software (Version 7.6.5, Tree Star Inc., Ashland, Oregon).

### Cell fractionation

Mitochondrial and cytoplasmic proteins were separated using a Mitochondria/Cytosol Fractionation Kit (Beyotime Inst. Biotech, Jiangsu, China, C3601) according to the manufacturer's protocol. Briefly, cells were harvested and washed twice with ice-cold PBS, cells were then incubated in 500 μl ice-cold mitochondrial lysis buffer on ice for 10 min. Cell suspension was then taken into a glass homogenizer and homogenized for 32 strokes using a tight pestle on ice. The homogenate was centrifuged at 800 g for 10 min at 4°C to remove any unbroken cells. The supernatant was further centrifuged at 8000 g for 10 min at 4°C to remove the mitochondrial fraction (pellet) and cytoplasmic proteins (supernatant). Proteins were quantified and subjected to immunoblotting.

### Statistics

2-tailed Student's t test was used for all statistical analyses, P < 0.05 were considered as significant difference.
